# Book Review: Gastrointestinal Physiology and Diseases Methods and Protocols

**DOI:** 10.3389/fcimb.2017.00319

**Published:** 2017-07-14

**Authors:** Somesh Baranwal

**Affiliations:** Centre for Biochemistry and Microbial Sciences, School of Basic and Applied Sciences, Central University of Punjab Bathinda, India

**Keywords:** inflammation, mouse model, colitis, ulcerative, imaging, barrier function

The gastrointestinal tract is one of the largest immunological organs and plays a pivotal role in maintaining normal homeostasis in the body. The statement made by Hippocrates more than 2,000 years ago “all disease begins in the gut” still holds true today. Studies in the past decades have revealed the importance of the gastrointestinal tract to human health. Defects in the digestive tract contribute to hundreds of medical conditions. Building upon the format of the Methods in Molecular Biology series, this book presents 29 protocols (divided into five parts) that mainly focus on the recent development of animal models to study gastrointestinal inflammation and disease progression.

*In-vitro* organoids have been shown to reflect a normal physiological state and have emerged as an excellent tool to study gastrointestinal physiology, development, and disease progression. The first part of the book provides a protocol for gene editing in mouse organoids using the CRISPR-Cas9 system. The subsequent chapter highlights a co-culture protocol that allows growth of mouse gastric organoids with immortalized stomach mesenchymal cells. This technique allows long-term *in-vitro* culture to study gastric cell physiology and host–pathogen interaction. The next chapter delineates a novel three-dimensional culture system that closely resembles the microenvironment of the intestinal stem cell niche and allows for long-term culture of primary gastrointestinal organoids for various studies. This section also covers tools to study HPLC and NMR-based metabolomics analysis of inflammatory bowel disease progression via the utilization of *in-vitro* cell-based assays and intestinal tissue samples. Additionally, two chapters in this section provide a protocol to measure *in-vivo* intestinal permeability using Ussing chambers and a protocol to quantify microRNA levels in intestinal epithelial cells.

The second part of the book covers tools for non-invasive *in-vivo* imaging of the gastrointestinal system. The first protocol describes a method to estimate reactive oxygen species generation and stem cell proliferation using candidate bacteria in the germ-free Drosophila intestine. The next protocol describes methods for visualization of *in-vivo* inflammatory hypoxia in the mouse gut via the use of hydroxyprobe staining and a non-invasive tool: the IVIS Spectrum imaging system. Using optical coherence tomography, one protocol in this section gives details about the non-invasive measurement of Eosinophilic Esophagitis (EoE) in a mouse model. The next protocol describes the use of near-infrared fluorescence endoscopy to visualize abnormal lesions developed during acute colitis. Moreover, it also illustrates a tool to measure *in-vivo* protein kinase activity in neutrophils from the inflamed intestine; transgenic mice expressing the FRET biosensor are utilized. The last protocol in this section discusses *in-vivo* imaging of myeloperoxidase, which is enriched in neutrophils, macrophages, and inflammatory monocytes.

The gut-associated lymphoid tissue (GALT) is filled with lymphocytes, macrophages, and other cells that participate in both innate and adaptive immune responses and are essential to maintain homeostasis in the intestine. Three chapters of the third section of the book are devoted to isolating macrophages, dendritic cells, and eosinophils from the mouse intestine. Emerging reports have proven a critical role for T regulatory cells (Treg) in autoimmune diseases such as inflammatory bowel disease and transplanted organ failure. Thus, one chapter elaborates methods to isolate natural T-regulatory (n-Treg) and induced T-regulatory (i-Treg) cells from the mouse intestine. Finally, one protocol in this section depicts a strategy to isolate Innate Lymphoid Cells (ILCs), which play a protective role in bacterial–host interactions.

Inflammatory bowel disease (IBD) is characterized by uncontrolled inflammation in the human intestine, which disrupts permeability and barrier function and causes ulceration in the gut. Animal models can play a crucial role in the study of basic mechanisms of IBD progression and preclinical testing of novel compounds. Thus, the fourth section of the book covers *in-vivo* tools to study gastrointestinal inflammation and injury. One chapter in this section discusses detailed methods to develop an *in-vivo* mouse model of Crohn's disease using tri-nitrobenzoic acid. Similarly, another chapter details oxazolone-induced colitis. Recent studies have provided compelling evidence concerning a role of the intestinal microbiota in the induction of colitis and cancer progression. One protocol in this section illustrates *Citrobacter rodentium*-induced infectious colitis in a murine model. Further, it also describes a Mongolian Gerbil model to study *Helicobacter pylori* induced inflammation and gastric cancer. Using zebrafish larvae, the last chapter of this section covers details to test novel compounds that protect against intestinal injury induced by the non-steroidal anti-inflammatory drug glafenine.

In the fifth and final section, two protocols detail induction of inflammatory colorectal cancer and colorectal cancer using Azoxymethane/Dextran Sodium Sulfate (AOM/DSS) and the Apc min/+ mouse model, respectively. Using the Apc min/+ mice, the role of environmental factor such as DSS and Sodium deoxycholate (a bile acid modifier) in the *in-vivo* modeling of colorectal cancer is also covered. The next protocol covers an *in-vivo* model to induce gastric adenocarcinoma via selective and reversible loss of parietal cells through use of an estrogen modifier, tamoxifen. The last chapter of the book covers a method to study oral cancer progression using the hamster buccal pouch (HBP) carcinogenesis model; this model uses 7,12-dimethylbenz[a]anthracene (DMBA) to closely recapitulate progression of human oral squamous cell carcinomas.

To summarize, this book is an excellent resource for researchers and physicians working in the area of gastrointestinal inflammation and cancer biology. These protocols will inspire the next generation of scientists that are interested in molecular biology and inflammation to better understand the complex interplay between the gastrointestinal microbiota and the human host, both of which play essential roles in maintaining gastrointestinal homeostasis.

## Author contributions

The author confirms being the sole contributor of this work and approved it for publication.

### Conflict of interest statement

The author declares that the research was conducted in the absence of any commercial or financial relationships that could be construed as a potential conflict of interest.

